# Intelligent diagnosis value of preoperative T staging of colorectal cancer based on MR medical imaging

**DOI:** 10.3389/fgene.2023.1119990

**Published:** 2023-02-16

**Authors:** Junqing Wang, Bingqian Chen, Jing Zhu, Junfeng Zhang, Rui Jiang

**Affiliations:** Department of Radiodiagnosis, General Hospital of Western Warfare Zone, Chengdu, Sichuan, China

**Keywords:** medical imaging technology, magnetic resonance imaging, colorectal cancer, preoperative T staging, intelligent diagnosis and treatment

## Abstract

Colorectal cancer is a common malignant tumor in clinic. With the change of people's diet, living environment and living habits, the incidence of colorectal cancer has risen sharply in recent years, which poses a great threat to people's health and quality of life. This paper aims to investigate the pathogenesis of colorectal cancer and improve the efficiency of clinical diagnosis and treatment. This paper firstly introduces MR Medical imaging technology and related theories of colorectal cancer through literature survey, and then applies MR technology to preoperative T staging of colorectal cancer. 150 patients with colorectal cancer admitted to our hospital every month from January 2019 to January 2020 were used as research objects to carry out the application experiment of MR Medical imaging in the intelligent diagnosis of preoperative T staging of colorectal cancer, and to explore the diagnostic sensitivity, specificity and histopathological T staging diagnosis coincidence rate of MR Staging. The final study results showed that there was no statistical significance in the general data of stage T1-2, T3 and T4 patients (p > 0.05); for patients with preoperative T stage of colorectal cancer, the overall diagnosis coincidence rate of MR Was 89.73%, indicating that it was highly consistent with pathological T stage; compared with MR Staging, the overall diagnosis coincidence rate of CT for preoperative T staging of colorectal cancer patients was 86.73%, which was basically consistent with the diagnosis of pathological T staging. At the same time, three different dictionary learning depth techniques are proposed in this study to solve the shortcomings of long MR Scanning time and slow imaging speed. Through performance testing and comparison, it is found that the structural similarity of MR Image reconstructed by depth dictionary method based on convolutional neural network is up to 99.67%, higher than that of analytic dictionary and synthetic dictionary, which proves that it has the best optimization effect on MR Technology. The study indicated the importance of MR Medical imaging in preoperative T staging diagnosis of colorectal cancer and the necessity of its popularization.

## 1 Introduction

### 1.1 Background and meaning

The recent changes in people’s diet, living environment, and living habits have also caused a series of malignant diseases such as colorectal cancer. Colorectal cancer is a malignant tumor disease common in middle-aged and elderly people. It is a type of disease in the digestive system and has a very high incidence in our country and the world. Because its early symptoms are hidden, it is easy to miss early diagnosis and treatment. Most patients have reached the middle and late stages when they are diagnosed, so they have a higher mortality and disability rate. Nowadays, MR medical imaging technology is widely used in clinical practice and has achieved good results (Xiao and Ding, 2019). This article’s goal is to examine the value of MR medical imaging in the accurate diagnosis of colorectal cancer’s preoperative T staging and to discover a successful method for early detection and treatment of colorectal cancer. Improving the diagnosis rate of patients will also improve their quality of life.

### 1.2 Related work

The incidence of early colorectal cancer is insidious, the clinical symptoms are not prominent, and there are many uncertainties with the increase of cancer. Because of the great harm of colorectal cancer to humans (Zhu et al., 2020), colorectal cancer has been studied very early, and many methods of diagnosis and treatment of colorectal cancer have been explored. So J S, Cheong C, and Oh S Y once stated that for patients with colorectal cancer, preoperative staging and application of various imaging techniques are of great significance to formulating treatment plans and predicting prognosis. For this reason, they discussed the use of CT technology to diagnose colorectal cancer (So et al., 2017). Xu Jiayi, Wang Jinkai and Zhou Lu discussed the value of serum C-reactive protein (CRP), sugar chain antigen 19-9 (CA19-9) and carcinoembryonic antigen (CEA) in the preoperative diagnosis of colorectal cancer (Xu et al., 2017). Ma K investigated the use of CT in the treatment of common malignant tumors including lung and colorectal cancer. They emphasized that while CT technology does not significantly contribute to the management of cholangiocarcinoma, it is helpful in the detection and management of colorectal cancer (Ma et al., 2018). Jaramillo FA and Daniel Upegui Jiménez proposed that CT colorectal cancer is the fourth leading cause of death in the world and the fifth leading cause of cancer death in Colombia. They believe that MRI is an ideal method to evaluate colorectal cancer, especially for screening, because it can be staged by determining the degree of invasion of the muscle layer and adjacent organs, which is useful for determining candidates for chemotherapy or preoperative radiotherapy and planning surgery procedure is crucial (Jaramillo and Upegui Jiménez, 2016). In addition, Park SH et al. stated that the preoperative colorectal tumor location is essential for proper resection and treatment planning. In response to the low positioning accuracy of traditional colonoscopy, they proposed to develop several new positioning techniques. They reviewed the tumor localization error rates of several preoperative endoscopic techniques, combined information about localization errors and risk factors for surgery-related adverse events, and concluded an effective method for accurately localizing colorectal tumors (Park et al., 2017). It can be seen from the above research results that although there are various methods for the diagnosis and treatment of colorectal cancer, there are still relatively few studies using MR medical imaging technology to intelligently diagnose the preoperative staging of colorectal cancer. Therefore, this article attempts to use this technology to explore the clinical intelligent diagnosis and treatment of colorectal cancer, with a view to adding a new treatment method to clinical treatment.

### 1.3 Innovations in this article

The innovations of this article are mainly reflected in the following aspects: 1) A malignant gastrointestinal tumor, colorectal cancer is exceedingly dangerous to people’s health and has a negative impact on patients’ quality of life. The clinical aspects of colorectal cancer are covered in this article. Methods of diagnosis and treatment greatly aid in the improvement of patient quality of life, the rate of early diagnosis, and the social practical importance and value; 2) The use of MR medical imaging technologies in the preoperative T staging of colorectal cancer is explored in this study. It is also suggested to apply deep learning algorithms to address the shortcomings of sluggish imaging speed and lengthy MR scanning times, which is crucial for enhancing and optimizing the performance of MR technology and enhancing its use in clinical diagnosis.

## 2 MR medical imaging technology and its intelligent diagnostic value for preoperative T staging of colorectal cancer

### 2.1 MR medical imaging

(1) MR

MR, or magnetic resonance, is a physical phenomenon related to the gravitational theory of magnetic fields (Joany and Logashanmugam, 2018). The principle is to combine the externally applied radio frequency energy field with the proton energy field of the human body. In a strong magnetic field environment, the proton can be thought of as a spin-nucleus system that can reflect certain aspects of the system by absorbing the externally delivered matching radio frequency energy field (Song et al., 2017; Guo et al., 2019).

(2) MRI

MRI, is magnetic resonance imaging, that is, magnetic resonance medical image. It is a type of magnetic resonance imaging-based imaging technology used for clinical disease diagnosis and treatment. When protons in the human body or the spin-nucleus system are magnetized to form a macroscopic magnetization vector, they will be driven by the radio frequency field. After being forced to return to its equilibrium position, the magnetization vector will keep circling the spin-nucleus system, eventually forming a closed coil. The coil will generate a magnetic resonance signal as a result of the nuclear system’s steady rotation. Since this kind of magnetic resonance signal cannot distinguish between the various positions of the spin nucleus, gradient fields must be applied in different directions of the spin nucleus. Through independent marking of its spatial position information during rotation, the spin nucleus is able to do so (Ali et al., 2019; Hoshino et al., 2019).

(3) Three-dimensional modeling of MR medical images

MR medical imaging uses the gravitational force of a magnetic field to treat the protons of the human body as a spin nucleus system, and absorbs the externally applied matching RF power field to generate magnetic resonance signals, creating a two-dimensional image in the process. The spatial position of the MR image will continue to consistently correspond to distinct pixel coordinates because of the nuclear system’s continual rotation (Yao et al., 2016; Pradhan et al., 2020). The patient’s lesion location is eventually localized in three dimensions using a three-dimensional picture. Transverse, coronal, sagittal, and any other cross-sectional images of the human body are included in general MR three-dimensional medical imaging (Acuna et al., 2017).

(4) MR image processing

Before they can be used effectively, medical photographs typically need to be cleaned, denoised, improved, and subject to other processes. This is because, during the picture capture process, the image will be susceptible to varying degrees of external interference, and the final image will be fuzzy or noisy (Lu and Wang, 2018). The magnetic field of the human body interacts with the radio waves of the MRI scanner to produce magnetic resonance images. Magnetic resonance signals are produced when the protons in the human body interact with radio wave energy. The coordinates of the various places of the tissues being investigated are represented by these signals. A tissue image of the human body is created by the fusion of many locations (Shakeel et al., 2020; Xu et al., 2022). We frequently employ image data processing technology for picture processing, and the procedure is as follows:

1) Image denoising and filtering. There are two widely used algorithms: the top-hat transform algorithm and the dual-tree complex wavelet transform algorithm.

The dual-tree complex wavelet transform approach comes first. On the basis of the complex wavelet, the dual-tree complex wavelet is produced. The formula for one-dimensional data transformation is:

$$\Phi \left(t\right)=\Phi_{h}\left(t\right)+i\Phi_{g}\left(t\right)$$ (1)

t is the time, $\Phi \left(t\right)$ is the total function.

When the complex wavelet becomes a dual-tree complex wavelet, its two-dimensional data transformation formula is:

$$\Phi \left(a,b\right)=\Phi \left(a\right)\Phi \left(b\right)$$ (2)

where $\Phi \left(a,b\right)$ is a two-tree complex wavelet. Second: the top-hat transformation method. Open and close the original image as follows:

$$\begin{array}{l} f\circ A=\left(f\Theta A\right)⊕A\\ f•A=\left(f⊕A\right)\Theta A \end{array}$$ (3)

Then the top hat transformation is as follows:

$$\begin{array}{l} WTH\left(a,b\right)=f\left(a,b\right)-f\circ A\left(a,b\right)\\ BTH\left(a,b\right)=f•A\left(a,b\right)-f\left(a,b\right) \end{array}$$ (4)

WTH (.) is the form of image transformation, and BTH (.) is the result of transformation.

2) Image augmentation. Typically, frequency domain and air domain methods are used.

First: To achieve the goal of boosting the contrast, the spatial technique involves processing the picture pixels in the space where the image is placed. The airspace method is expressed as follows:

$$g\left(a,b\right)=U\left[f\left(a,b\right)\right]$$ (5)

g (a, b) is the image pixel in space, and U is the transformation function.

Second: The image is first transformed by the frequency domain method, the parameters are then improved, and lastly the enhanced image is changed back to the original region (Guo et al., 2018).

(6) Advantages and disadvantages of magnetic resonance imaging

At present, in addition to MR, the commonly used medical diagnostic imaging techniques in clinical use include CT and X-ray. X-ray is the earliest and most common medical imaging technology. It has strong penetrability and is suitable for imaging examinations of high-density tissues. It is generally used for bone examination, but the imaging is not clear and will produce harmful radiation to the human body. CT images are clearer than X-ray images and can be used to examine internal organs and brain tissue, but the radiation it produces is more harmful than X-rays. In contrast, MR not only has a high-definition resolution, but also does not generate harmful radiation to the human body. It can also perform arbitrary slices in different directions according to the patient’s different body positions. However, MR images also have a shortcoming that cannot be ignored. The equipment takes a long time to scan, and the inspection of one part often takes a long time, which not only aggravates the patient’s pain, but also increases its economic burden (Hu et al., 2020; Monroe et al., 2021). Therefore, in order to improve this problem and improve the speed and efficiency of MR image scanning, this article attempts to optimize and improve MRI. This content will be specifically introduced in the third section with colorectal cancer as an example.

### 2.2 Colorectal cancer

(1) The symptoms of colorectal cancer

Colorectal cancer, also known as colorectal cancer, is a common malignant tumor of the digestive tract, including colon cancer and rectal cancer. Its lesions often occur in the colorectal epithelial tissue. The high incidence of colorectal cancer is mainly middle-aged and elderly people between 54 and 81 years old, mostly in developed countries. At present, the pathological mechanism of colorectal cancer is not yet clear, but it is closely related to factors such as people’s diet, living environment, family hereditary polyps and chronic inflammation. Studies have shown that people who eat high-fat, high-calorie, low-fiber foods are more likely to develop colon cancer (Yang et al., 2019). China has long been a country with a low incidence of colorectal cancer, but with the improvement of living conditions, people’s diet and living conditions have undergone significant changes, and the incidence of colorectal cancer has gradually increased.

1) The early stage of the disease is difficult to detect, and the symptoms are extremely hidden, which often leads to missed and misdiagnosed phenomena. Many patients are often in the late stage of the disease when they are diagnosed. Therefore, the disease has a high mortality and disability rate;
2) The elderly are a frequent group of the disease, which is extremely harmful to the quality of life and health of the elderly;
3) The tumor cells in the lesion are prone to metastasis, which affects the normal physiological functions of the surrounding organs and other parts of the body;
4) Postoperative complications are obvious and difficult to cure, and the quality of life of patients has significantly decreased (Sun et al., 2017).
(2) Clinical manifestations of colorectal cancer
1) Early clinical symptoms. In the early stages of the disease, the symptoms of colorectal cancer are not yet obvious. However, when the tumor in the intestine grows larger, the patient’s bowel habits will gradually change, showing symptoms such as bleeding stool, diarrhea, alternating diarrhea and constipation, and local abdominal pain. The frequency of excretion increases, accompanied by a small amount of mucus and blood in the stool.
2) Middle and late clinical symptoms. In the middle and late stages, the tissues and organs around the colorectal have different degrees of necrosis and changes, and their functions are obviously impaired. For example, the surrounding tissues such as the bladder and prostate will have symptoms such as frequent urination, urgency and difficulty urinating. In severe cases, the tumor cells of colorectal cancer will also move and metastasize to distant tissues, such as liver and lungs. Patients with advanced colorectal cancer may also experience weight loss, loss of appetite, and anemia (Que, 2018).
(3) Preoperative T staging of colorectal cancer

T stage: The tumors located in the local intestinal mucosa were divided into T1, T2, T3 and T4 according to the depth of invasion. T4 suggested that the surrounding structures and tissues were invaded. The larger the number, the later the stage. Preoperative T staging is a preoperative preparation for the treatment of gastrointestinal diseases. Colorectal cancer is a malignant gastrointestinal tumor, and surgery is quite risky, coupled with the special physiological structure and function of the human rectum. Therefore, in order to increase the success rate of surgery and reduce the risk of surgery, it is very important to determine and stage the condition of colorectal cancer patients before surgery. The more accurate the preoperative staging is, the better it is for patients to undergo adjuvant chemotherapy before surgery, which can reduce the stage of malignant tumors, and also facilitate the selection and arrangement of surgical plans, thereby achieving the purpose of improving the success rate of surgery and the survival rate of patients. It is also very effective for the prognosis of patients, reducing the recurrence rate of colorectal cancer patients after surgery and greatly improving their quality of life (Frank et al., 2018).

### 2.3 Value of MR medical imaging on the intelligent diagnosis of preoperative T staging of colorectal cancer

With the development of medical technology, X-ray, CT and MR technologies have gradually been widely used in the medical field. Although MR has advantages over X-ray and CT in terms of imaging quality and impact on the human body, long scanning time and slow imaging speed are also its biggest disadvantages. High-quality images provide more accurate positioning for the preoperative staging of colorectal cancer. This article attempts to use colorectal cancer as a research sample to reconstruct its MR images by improving and optimizing MR imaging technology (Fan et al., 2017). Here, we will use the concept of deep learning to improve MR technology through deep learning algorithms, and use three different dictionary learning methods to reconstruct MR images. Deep dictionary method based on convolutional neural network can better process the structural details in the image to produce clearer results when training the convolutional neural network model.

(1) Reconstruction of magnetic resonance images using a parsing dictionary

$$TV\left(v\right)=\iint \nabla v\left(x,y\right){\|}_{1}dxdy=\iint \left|\frac{\partial v}{\partial x}\right|+\left|\frac{\partial v}{\partial y}\right|dxdy$$ (6)

$$TV\left(v\right)={\iint \left\|\nabla v\left(x,y\right)\right\|}_{2}^{2}+\Phi^{2}dxdy={\iint \left(\frac{\partial v}{\partial x}\right)}^{2}+{\left(\frac{\partial v}{\partial y}\right)}^{2}+\Phi^{2}dxdy$$ (7)

The MRI image is optimized and solved using the norm of total variation, as in formula (8)

$$\min_{x}{\left\|F_{u}x-y\right\|}_{2}^{2}+\lambda TV{\left(x\right)}_{1}$$ (8)

(2) Reconstruction of magnetic resonance images using a synthetic dictionary

In contrast to the analytical dictionary’s quick and easy computation approach, the synthetic dictionary can describe more complicated images, has some adaptability, and can lessen the noise and filtering issues that the analytical dictionary has because of down sampling (Sibertinblanc et al., 2016). Here, we focus primarily on introducing the sparse representation, dictionary building, and synthetic dictionary reconstruction processes.

A transformation matrix created using the corresponding points of the magnetic resonance signal is the so-called dictionary. The magnetic resonance image’s sparse representation is

y=Dα (9)

The sparse coefficient is represented by a, while D is the dictionary matrix.

Create a synthetic dictionary after that, using the formula for the mathematical model (10)

$$\min_{D,X}{\left\|Y-DX\right\|}_{2}^{2},s,t,\forall i,{\left\|x_{i}\right\|}_{0}\leq T$$ (10)

Split the model into a sparse representation problem of a single sample, and get:

$$\min_{x_{i}}{\left\|y_{i}-Dx_{i}\right\|}_{2}^{2},s,t,\forall i,{\left\|x_{i}\right\|}_{0}\leq T$$ (11)

(3) Deep dictionary-based magnetic resonance image reconstruction

Convolutional neural network algorithms are combined in this deep dictionary, which reconstructs MRI images by utilizing the powerful flexibility and self-learning capabilities of ANNs. We previously presented two strategies for learning dictionaries. While the synthetic dictionary has some adaptability, its ability to denoise and filter the image is superior. The analytical dictionary is a fixed transformation that can only handle a few simple image changes, and its sparse expressiveness is weak. Strong, however the reconstructed image effect is unsatisfactory when there is less image data (Park and Lee, 2017). So, we once more suggest a convolutional neural network-based deep dictionary approach.

$$H\left(R,b;Px,y\right)=\frac{1}{2}{\left\|h_{r,b}\left(x\right)-y\right\|}^{2}$$ (12)

which represents the cost function, represents the connection parameters between the layers. Assuming a data set containing n samples, the overall cost function is:

$$\begin{array}{ll} H\left(W,b\right)=\left(\frac{1}{n}\sum_{i=1}^{n}H\left(W,b;x^{\left(i\right)},y^{\left(i\right)}\right)\right)+\frac{\lambda }{2}{\sum_{l=1}^{n_{l}-1}\sum_{j=1}^{s_{l}+1}\left(W_{ji}^{l}\right)}^{2} & =\left[\frac{1}{m}\sum_{i=1}^{m}\left(\frac{1}{2}{\left\|h_{w,b}\left(x^{\left(i\right)}-y^{\left(i\right)}\right)\right\|}^{2}\right)\right]+\frac{\lambda }{2}\sum_{l=1}^{n_{l}-1}\sum_{i=1}^{s_{l}}\sum_{j=1}^{s_{l}+1}\left(W_{ji}^{l}\right) \end{array}$$ (13)

The cost function is iterated by the dimensionality reduction method, and then

$$W_{ij}^{l}=W_{ij}^{l}-\alpha \frac{\partial }{\partial w_{ij}^{l}}H\left(W,b\right)$$ (14)

$$b_{i}^{l}=b_{i}^{l}-\alpha \frac{\partial }{\partial b_{i}^{l}}H\left(W,b\right)$$ (15)

Among them, α represents the rate of learning.

## 3 Application experiment of MR medical imaging in the intelligent diagnosis of preoperative T staging of colorectal cancer

### 3.1 General information collection

In order to specifically explore the application value of MR medical imaging in the preoperative T-analysis intelligent diagnosis of colorectal cancer, this article collected 150 colorectal cancer patients admitted to our hospital from January 2019 to January 2020. All patients were admitted to the hospital. Routine examinations were performed without any preoperative treatment. MR examinations were performed before the operation. Patients who could not tolerate MR technical examinations and surgical contraindications were excluded. Finally, 146 effective cases were selected, including 86 males and 60 females; age 38–73 years old, the average age is 56 ± 2.45 years, and the average age of illness is 14 ± 3.56 years. The general information of the patient is shown in Table 1.

**TABLE 1:** General information of patients.

Group	Man	Woman	Average age	Average age of disease
T1-2	27	14	51 ± 2.14	9 ± 2.17
T3	34	24	54 ± 3.22	7 ± 3.16
T4	27	20	53 ± 2.57	11 ± 2.34

### 3.2 MR inspection method

After grouping every patient in accordance with the preoperative T staging, they were subjected to preoperative MR technical examination to collect colorectal tumor cell images. The night before the examination, all patients took bowel cleansing drugs and fasted food and water. Use our own MRI scanner for operation, with 16-channel body phased array coils. The parameters of the scanner are: pulse sequence repetition time 2600–3400 ms, echo time 150 ms, field of view 400 mm, the thickness is 5 mm, the layer spacing is 1 mm, the matrix is 260 × 298, the number of excitations is 3, and the flip angle is 90°. First, the patients in each group were injected with 0.1 mmol/kg gabapentin meglumine as a contrast agent by intravenous injection, and kept advancing at a speed of 2 ml/s. MR scanning was performed immediately after the injection was completed. At the same time, the patient was instructed to keep breath-hold during the scan, first scan at a uniform speed, and then strengthen the local scan until a complete colorectal image is obtained.

### 3.3 Staging standards and observation indicators

Stage T1-2: there are obvious gaps in the fat at the lesion of the colorectal intestinal wall. Even the enhanced scan shows that the outer edge of the intestinal wall is smooth and there is no sign of nodules. T3: fat around the lesion on the intestinal wall. There are sparse markings, and the muscle layer has been invaded to a certain extent. When the scan is increased, the outer edge of the intestinal wall is uneven, and nodules are slightly prominent; stage T4: there are no gaps in the fat around the lesion of the intestinal wall and the adjacent tissues and organs. The boundary is blurred during enhanced scanning. The tumor has seriously invaded the colorectal fascia and surrounding organs.

According to the results of MR examination, the degree of invasion of each layer of the colorectal wall is evaluated, and the sensitivity, specificity, and rate of diagnostic coincidence for each colorectal cancer stage are assessed in comparison to the histological T staging.

### 3.4 Data processing

The data processing and analysis of this experiment used SPSS 22.0 statistical analysis software. The MR staging results of colorectal cancer and the pathological T staging results were tested for variance and chi-square, and the sensitivity, specificity and diagnostic coincidence rate of MR staging were compared. Among them, *p* > 0.05 indicates no significant statistical difference, *p* < 0.05 indicates significant statistical difference, *p* < 0.01 indicates extremely significant statistical difference; Chi-square 1 indicates high degree of agreement, 2 indicates general agreement, 3 indicates a low degree of compliance.

## 4 Discussion of experimental results

### 4.1 Comparison of differences in general patient information

**FIGURE 1:**
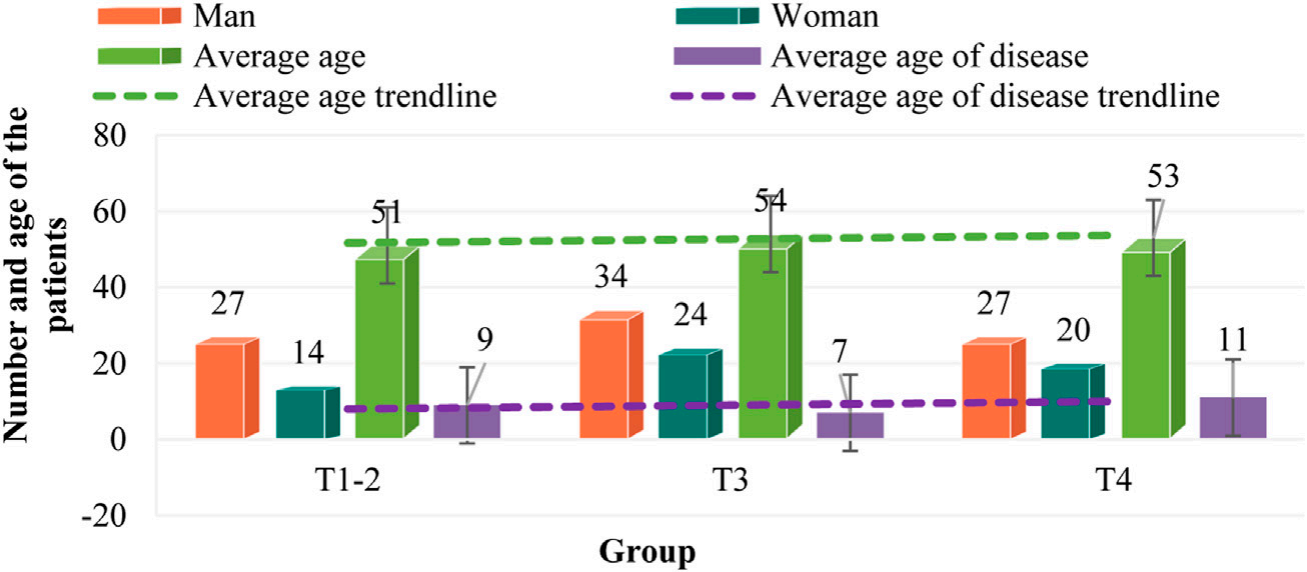
Comparison of general patient information.

According to the general data of colorectal cancer patients collected in the third part, the general conditions of each group of patients are counted, and the *p*-value and chi-square value of each group of patients on gender, age and disease age are calculated, and plotted as shown in Figure 1. The histogram to compare the differences in the general conditions of patients in each group.

As can be seen from Figure 1, in the samples collected in T1-2, there are 27 men and 14 women, with an average age of 51 years, and the average age of patients is 42 years. In the samples collected in T3, there are 34 men and 24 women, with an average age of 54 years, and the average age of patients is 47 years. In T4, there are 27 men and 20 women, with an average age of 53 years, and the average age of patients is 42 years. There was no significant difference in general information among T1-2, T3 and T4 patients (*p* > 0.05). The age of patients in each group is 50–60 years old, and the age of onset is about 10 years. The majority of men and women are men. Among the three groups, the number of patients in T3 group was the largest, with 58 cases, followed by T4 group with 47 cases, and T1-2 group with 41 cases.

### 4.2 Comparison of results between MR staging and pathological T staging

To ascertain the sensitivity, specificity, and diagnostic coincidence rate of MR staging, the patients’ preoperative T staging was performed post-experimentally in accordance with the findings of the MR scan. The results were then compared with the pathological T staging performed prior to the experiment.

(1) Comparison of results between MR staging and pathological T staging

**FIGURE 2:**
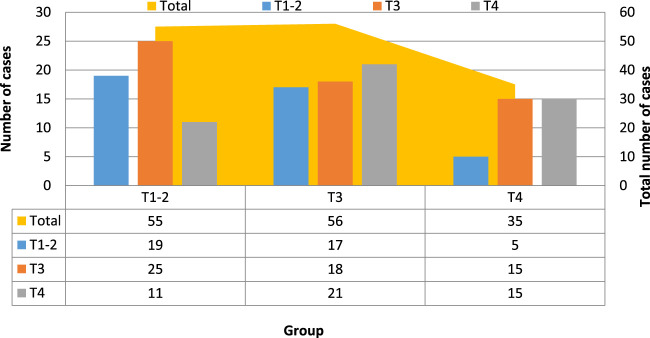
Comparison of MR staging and pathological T staging.

The results of MR staging and pathological T staging are compared as shown in Table 2 and Figure 2.

**TABLE 2:** Comparison of MR staging and pathological T staging.

Pathological staging MR staging				T1-2	T3	T4	Total
T1-2				19	25	11	55
T3				17	18	21	56
T4				5	15	15	35
Total	41	58	47	146			

Table 2 and Figure 2 show that the pathological T staging detected and diagnosed by MR technology differs in certain ways from the T staging (*p* < 0.05). According to the pathological T staging, 146 patients were divided into T1-2 stages. 41 cases, 58 cases in T3 stage, 47 cases in T4 stage, and after MR examination, the T stage of the patients was 55 cases in T1-2 stage, 56 cases in T3 stage, and 35 cases in T4 stage. This shows that the symptoms of 14 patients were overestimated, of which 12 were misestimated as T4 stage, and 2 were misestimated as T3 stage. These results show that the steps and contents of the study need to be more specific, so this is only an individual case.

(2) Evaluation of the sensitivity, specificity, and diagnostic coincidence rate of MR staging

**FIGURE 3:**
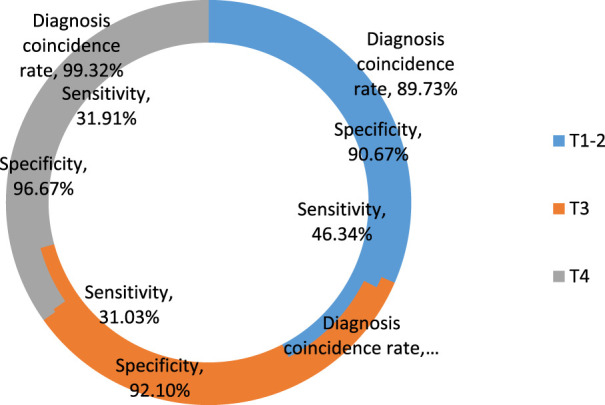
Sensitivity, specificity and diagnostic coincidence rate of MR staging.

The findings, which are shown in Table 3 and Figure 3, include evaluations of the sensitivity, specificity, and rate of diagnostic coincidence for MR staging as well as comparisons between pathological T staging results and MR staging results.

**TABLE 3:** Diagnostic coincidence rate, sensitivity, and specificity of MR staging.

	Sensitivity	Specificity	Diagnosis coincidence rate	*p*	X2
T1-2	46.34% (19/41)	90.67% (68/75)	89.73% (131/146)	0.021	0.85
T3	31.03% (18/58)	92.1% (70/76)	95.89% (140/146)	0.025	0.83
T4	31.91% (15/47)	96.67% (87/90)	99.32% (145/146)	0.042	0.81

In Table 3, in T1-2 phase, the sensitivity, specificity and coincidence rate of MRI were 46.34%, 90.67% and 89.73% respectively. In T3 phase, their sensitivity, specificity and coincidence rate were 31.03%, 92.1% and 95.89% respectively. In T4 phase, their sensitivity, specificity and coincidence rate were 31.91%, 96.67% and 99.32% respectively.

From Table 3 and Figure 3, it can be seen that the total diagnostic coincidence rate of MR for preoperative T staging of colorectal cancer patients is 89.73%, the sensitivity of each stage is 46.34%, 31.03% and 31.91%, and the specificity is 90.67%, 92.1% and 96.67%, *p*-values were 0.021, 0.025, and 0.042, respectively, and the chi-square values were all greater than 0.8, which indicates that the preoperative T staging of MR and the pathological T staging have a higher degree of agreement, and the agreement and consistency strong.

### 4.3 Comparison of results between CT staging and pathological T staging

**FIGURE 4:**
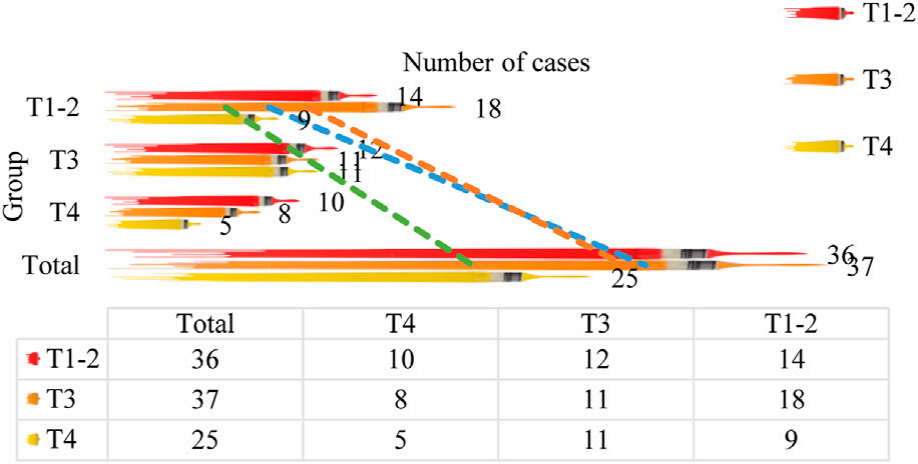
Comparison of CT staging and pathological T staging results.

In order to further explore the application value of MR technology in the intelligent diagnosis of colorectal cancer preoperative T staging, and at the same time apply CT technology to the preoperative T staging diagnosis of colorectal cancer, compare the difference between CT staging and pathological T staging, and it is compared with MR staging to analyze the advantages of MR staging and CT staging. In addition, 98 patients with colorectal cancer admitted to our hospital were selected. The T stages were 36 cases in T1-2 stage, 37 cases in T3 stage, and 25 cases in T4 stage. According to the operation steps of MR examination, these 98 patients were examined by CT, and finally the CT staging was obtained, and the results were compared with the pathological T staging, as shown in Table 4 and Figure 4.

**TABLE 4:** Comparison of CT staging and pathological T staging results.

Pathological staging CT staging	T1-2	T3	T4	Total
T1-2	14	18	9	41
T3	12	11	11	34
T4	10	8	5	23
Total	36	37	25	98

Table 4 and Figure 4 demonstrate that there are several differences between the T staging and the pathological T staging detected and diagnosed by CT technology (*p* < 0.05). According to the pathological T staging, 98 patients were divided into T1-2 stages. There were 36 cases in T3 stage, 37 cases in T4 stage, 25 cases in T4 stage. After CT examination, the T stage of patients was 41 cases in T1-2 stage, 34 cases in T3 stage, and 23 cases in T4 stage. This shows that the symptoms of five patients were overestimated, of which 3 were overestimated as T3 stage, and 2 were overestimated as T4 stage.

**FIGURE 5:**
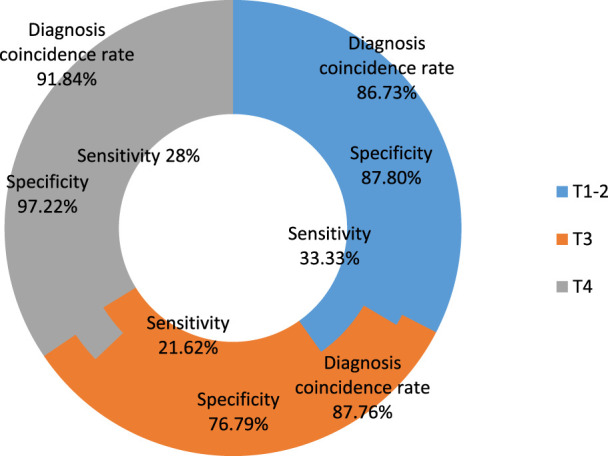
CT staging’s sensitivity, specificity, and diagnostic coincidence rate.

The *p*-value and chi-square value of the two were determined in accordance with the distinction between CT staging and pathological staging, and the sensitivity, specificity, and diagnostic coincidence rate of CT staging were examined. Table 5 and Figure 5 present the findings.

**TABLE 5:** CT staging’s sensitivity, specificity, and rate of diagnostic concordance.

	Sensitivity	Specificity	Diagnosis coincidence rate	*p*	X2
T1-2	33.33% (12/36)	87.8% (36/41)	86.73% (85/98)	0.036	0.75
T3	21.62% (8/37)	76.79% (43/56)	87.76% (86/98)	0.026	0.79
T4	28% (7/25)	97.22% (70/72)	91.84%90/98)	0.038	0.81

Table 5 and Figure 5 show that the overall CT diagnostic coincidence rate for colorectal cancer patients’ preoperative T staging is 86.73%, the sensitivity of each stage is 33.33%, 21.62% and 28%, and the specificity is 87.8%, 76.79% and 97.22%, the *p*-values were 0.036, 0.026 and 0.038, and the chi-square values were all less than 0.8. This shows that although the diagnosis of CT staging and pathological T staging has a significant statistical difference, the agreement is average. Compared with MR staging, its agreement is slightly lower, which proves that MR technology is useful for preoperative T staging of colorectal cancer.

### 4.4 Effect of optimization of MR technology using various deep learning algorithms

According to the foregoing, among the existing clinical auxiliary diagnostic imaging technologies, although many studies have shown that MR technology is the most effective in auxiliary diagnostics, it also has serious problems such as long scanning time and slow acquisition of data and images. This article attempts to improve and optimize its core steps based on the working principle of MR technology. In order to improve the reconstruction of colorectal cancer pictures obtained by MR, three distinct dictionary learning methods are proposed (The complete calculation procedure and deduction steps refer to Section 2.3 of this article). In this section, we will compare the differences in peak signal-to-noise ratio and structural similarity of the reconstructed MR images produced by three different dictionary learning algorithms, and identify the algorithm that produces the best image reconstruction quality and the quickest imaging speed.

(1) Results of analytic and synthetic dictionary optimization

To sample MR pictures, use analytical dictionary and synthetic dictionary learning methods. Set the sampling rates to 20%, 30%, 40%, and 50% depending on the desired sampling rate. Under the same sample rate background, compare the two. Different learning is used to determine the reconstructed image’s peak signal-to-noise ratio and structural similarity. Table 6 display the statistical findings. Analytical dictionary is 32.25% when sampling rates are 30%.

**TABLE 6:** Effect of optimization of MR technology using various deep learning algorithms.

Learning algorithm		30%	40%	50%	60%
Analytic dictionary	Peak signal to noise ratio(A)	32.25	36.29	38.47	41.56
	Structural similarity(A)	0.8521	0.8735	0.9174	0.9387
Synthetic dictionary	Peak signal to noise ratio(S)	33.54	37.55	41.45	44.67
	Structural similarity(S)	0.8741	0.9145	0.9279	0.9536

Table 6 shows that the peak signal-to-noise ratio and structural similarity of the synthetic vocabulary are higher than the values of the analytical dictionary under the same sampling rate, demonstrating that the synthetic dictionary has a superior influence on picture reconstruction.

(2) The optimization result of the deep dictionary

The deep dictionary algorithm we suggested is built on a convolutional neural network, based on the aforementioned. Sample training and testing are necessary depending on the neural network’s properties. Select the first 200 MR images as the training group, the last 100 as the test group, and 300 MR images as the sample data. With 50, 100, 150, 200, 250, and 300 samples, respectively, iterative training and testing are conducted. Each sample interval is recorded. The results are displayed in Table 7 compare the peak-to-noise ratio and structural similarity of the image. They also compare the difference between the *p*-value and the chi-square value of each sample interval.

**TABLE 7:** Optimization results based on deep dictionary.

	50	100	150	200	250	300
Peak signal to noise ratio	34.47	34.47	36.55	41.89	42.38	45.54
Structural similarity	0.9118	0.9357	0.9472	0.9551	0.9678	0.9967
*p*-value	0.256	0.478	0.351	0.571	0.610	0.617
X2	0.897	0.880	0.857	0.872	0.810	0.835

Table 7 show that when the sample size grows, the quantitative evaluation value of the deep dictionary-reconstructed image rises, showing a greater effect. The structural similarity also shows that the deep dictionary-reconstructed image is superior to the prior image. The two algorithms produce images that are more faithful to real MR scans. The structural similarity value reaches 0.9967 when the sample size is 300, which is significantly higher than the values obtained by the first two techniques. The sample training results are compatible with the test results (X2 > 0.8) and there is no statistically significant difference between the sample intervals, as can be observed from the *p*-value and Chi-square value (*p* > 0.05). As can be observed, the MRI picture generated by the deep dictionary approach based on the convolutional neural network has the best quality and the fastest imaging speed of the three dictionary learning methods.

## 5 Conclusion

Colorectal cancer is a malignant tumor disease that threatens human health and quality of life, especially among middle-aged and elderly people. Because its early symptoms are hidden and difficult to detect, its death and disability rates are high. Preoperative T staging of colorectal cancer is important for creating a precise surgical treatment plan, which is important for enhancing patient quality of life and increasing long-term survival rates.

MR is a cutting-edge imaging technique used in clinical and auxiliary medicine. In individuals with colorectal cancer, it is crucial to examine the tumor’s location, its depth, and its connections to other tissues. Compared to CT and conventional X-rays, it offers additional benefits. Rectal cancer preoperative T staging offers significant diagnostic significance.

This study confirmed through experiments that MR has higher sensitivity, specificity and consistency in the diagnosis of each stage than CT. At the same time, it makes an algorithm based on convolutional neural network and compares the performance tests of analytic, synthetic, and deep dictionaries. A deeper lexicon is better for optimizing MR and is better for enhancing MR scanning and imaging speed. However, this article is not very proficient in the clinical diagnosis and treatment of colorectal cancer and needs further improvement. This is very helpful to increase the early diagnosis rate and improve the quality of life of patients. The inadequacy of the article is that it does not explain the situation of patients in each T stage, and how to care for patients in each T stage. The results of each stage are still not enough. I hope that we can strengthen, guide and formulate treatment plans and evaluate the prognosis in the future.

## Data Availability

The original contributions presented in the study are included in the article/supplementary material, further inquiries can be directed to the corresponding author.
